# Bulla Formation and Tension Pneumothorax in a Patient with COVID-19

**DOI:** 10.4269/ajtmh.20-0736

**Published:** 2020-07-08

**Authors:** Kosuke Yasukawa, Arathy Vamadevan, Rosemarie Rollins

**Affiliations:** Division of Hospital Medicine, Department of Medicine, MedStar Washington Hospital Center, Washington, District of Columbia

**Figure 1.**
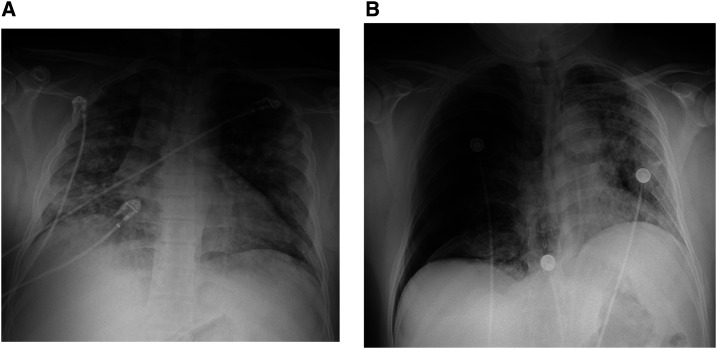
(**A**) Patchy airspace disease scattered throughout both lungs with a peripheral predominance. (**B**) A large right pneumothorax with leftward shift of mediastinal structures and re-demonstration of patchy airspace opacities throughout both lungs.

A 37-year-old man with no significant past medical history presented to the emergency department with a 4-day history of nonproductive cough and shortness of breath. A chest X-ray showed bilateral infiltrates with a peripheral predominance (Figure 1A). Polymerase chain reaction was positive for SARS-CoV-2. The patient developed worsening respiratory distress, was transferred to the intensive care unit, and was placed on a high-flow nasal cannula. He received a course of remdesivir and convalescent plasma therapy. A repeat chest X-ray on day three showed findings similar to those on the initial chest X-ray. His respiratory status improved, and he was discharged on day 12.

**Figure 2.**
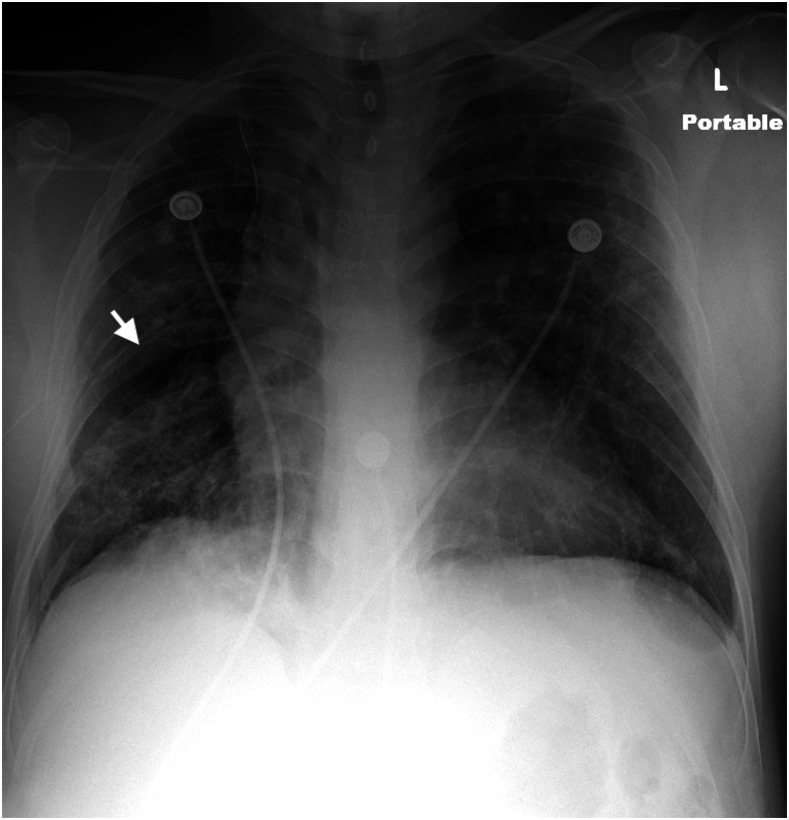
An air-filled bulla is seen lateral to the right hilum (arrow). There is a small residual right pneumothorax following right chest tube placement.

**Figure 3.**
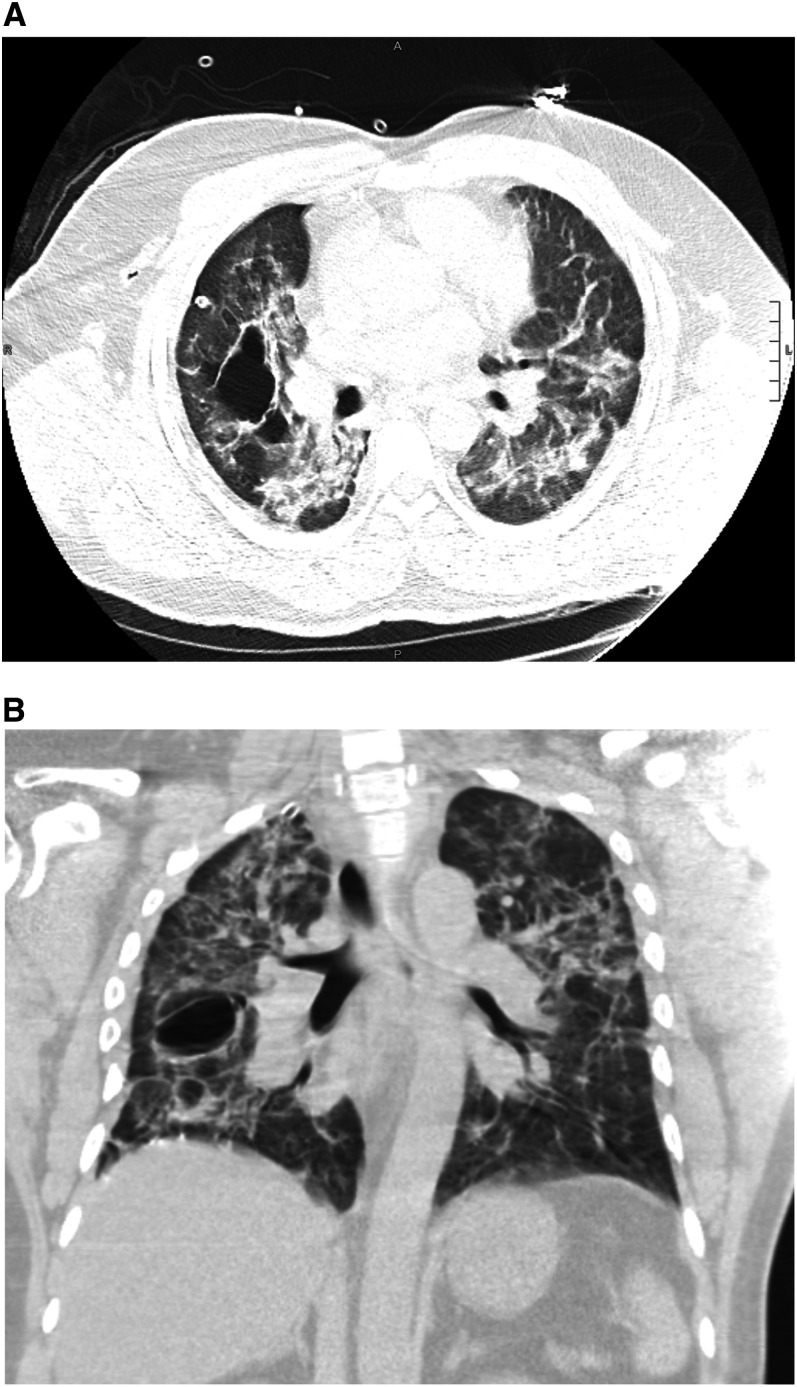
Computed tomography of the chest demonstrating extensive bilateral infiltrates consistent with COVID-19 pneumonia and a right mid-lung bulla measuring 5.6 cm (anteroposteriorly) by 3.3 cm transversely by 2.7 cm craniocaudally. Transverse view (**A**) and coronal view (**B**).

He returned to the emergency department after 14 days complaining of right-sided pleuritic chest pain and shortness of breath of approximately 24-hour duration. A chest X-ray demonstrated a large right pneumothorax with a leftward shift of the mediastinal structures consistent with a tension pneumothorax (Figure 1B). A 16-French thoracostomy tube was emergently placed. A repeat chest X-ray showed the presence of bulla lateral to the right hilum (Figure 2). A subsequent chest computed tomography (CT) demonstrated extensive bilateral infiltrates and a right mid-lung bulla (Figure 3A and B). He remained stable, serial chest X-rays showed diminishing size of the pneumothorax, the chest tube was removed after 5 days, and the patient was discharged.

Although alveolar rupture due to barotrauma can occur in the setting of invasive mechanical ventilation, there are sporadic reports of spontaneous pneumothorax occurring in patients with COVID-19 who did not require invasive mechanical ventilation.^1–3^ Two cases of tension pneumothorax have been reported in non-intubated patients with COVID-19. Similar to Flower et al.’s case, our patient also had a bulla. In our patient, the bulla was not noted on the chest X-ray from the initial admission, indicating formation secondary to his COVID-19 pneumonia. Radiologic studies have shown that patients with COVID-19 pneumonia can develop cystic changes during the course of SARS-CoV-2 infection.^4,5^ Sun et al.^1^ reported a formation of a giant bulla and subsequent pneumothorax in a patient with COVID-19. The pathophysiology of cystic changes and bullae formation in COVID-19 is still unknown. Further studies are needed to evaluate the long-term pulmonary consequences of COVID-19 pneumonia and the risk of pneumothorax in patients who recover from the initial acute respiratory failure. The utility of follow-up chest imaging to evaluate bulla formation and other structural changes needs to be investigated.

In conclusion, bulla formation and spontaneous pneumothorax is a possible complication of COVID-19. Spontaneous pneumothorax should be considered in a patient with COVID-19 pneumonia who develops chest pain or acute worsening of dyspnea.
